# Efficacy of Double Membrane Filtration Immunoadsorption in Severe C1q-Binding Donor-Specific Antibody-Positive Acute Humoral Kidney Allograft Rejection: A Case Series

**DOI:** 10.1159/000528748

**Published:** 2023-04-12

**Authors:** Martin Russwurm, Tanja Maier-Giebing, Birgit Kortus-Goetze, Philipp Russ, Hermann-Josef Groene, Joachim D. Hoyer

**Affiliations:** ^a^Division of Nephrology, Centre for Internal Medicine, University Hospital Marburg, Marburg, Germany; ^b^Institute of Pharmacology, Philipps-University, Marburg, Germany; ^c^Medical Faculty, University of Heidelberg, Heidelberg, Germany

**Keywords:** Kidney transplantation, Acute antibody-mediated rejection, C1q, Double membrane filtration, Immunoadsorption

## Abstract

**Introduction:**

Acute antibody-mediated rejection (ABMR) is an important threat to renal allograft survival in the early transplant period and the major single cause of graft loss in the first postoperative year. Semi-selective immunoadsorption (IA) remains one of the commonly applied treatments in ABMR, reducing allo-reactive antibody load. Adding double filtration plasmapheresis (DFPP) to IA might enhance therapeutic efficacy by also addressing innate humoral effectors like complement factors.

**Methods:**

Four patients with ABMR were treated with DFPP + IA. Clinical, histological, and immunological data and adverse events were retrospectively collected.

**Results:**

Here we present four high-risk treatment-refractory ABMR cases with C1q-binding donor-specific antibodies and histology of humoral rejection under treatment with DFPP + IA. While the earlier cases (within the first year after transplantation) showed marked reduction in ABMR severity and improvement of kidney function, the later cases did not respond accordingly. Late ABMR patient 1 stabilized, whereas late ABMR patient 2 did not respond to treatment.

**Conclusions:**

Our data support the consideration of DFPP + IA as a rescue treatment option in early, severe, high-risk ABMR cases in which other treatments failed.

## Introduction

Acute humoral rejection (acute antibody-mediated rejection [ABMR]) is a major threat to renal allograft survival in the early transplant period. Despite its relatively low incidence of approximately 1–3%, it accounts for up to 20–40% of graft losses within the first year [1].

The proposed pathological hallmark of ABMR is the development of antibodies against the allograft (donor-specific antibodies [DSAs]) with subsequent complement activation and influx of innate immune effector cells, foremostly NK cells and monocytes [2]. The importance of DSA as a biomarker in acute allograft rejection is well recognized [3, 4]. The role and potential detrimental effect of antibodies capable of binding to complement were highlighted in a study by Loupy and others, in which complement factor C1q-binding DSA in comparison to non-C1q-binding DSA displayed an independent risk factor for graft loss [5].

Although various immunomodulatory and -suppressive medications and apheresis techniques are used to treat or avert ABMR [6, 7, 8, 9, 10], there is no gold standard for treatment mainly due to lack of thoroughly designed randomized controlled trials or highly selected study cohorts. Commonly used medical treatments for ABMR (pre-emptively and therapeutically) are implemented to inhibit complement activation or assembly (C5b inhibitor [11], C1 esterase inhibitor [12]), suppress adaptive immune effector cells (anti-thymocyte globulin [13], CD20 antibody [14]), and to cleave or deplete antibodies (immunoglobulin G-degrading enzyme of *Streptococcus pyogenes* [15], intravenous immunoglobulins [IVIG] [16]). Extracorporeal plasma treatments are primarily implemented to reduce serum humoral effectors. It could be demonstrated that the reductive capacity of a combined treatment of double filtration plasmapheresis (DFPP, also referred to as membrane filtration MF) and immunoadsorption (IA) resulted in more profound depletion of serum IgM and complement factor C1q than IA alone. This approach might thus be of further importance in attenuating the self-perpetuating inflammatory process of ABMR in a pre-emptive manner, as was shown by Eskandary and colleagues [17].

Here we report on four patients with de novo C1q-binding DSA and biopsy-proven treatment-refractory ABMR who were treated with DFPP + IA in a compassionate use intent. It was surmised that DFPP + IA is not only capable of reducing ABMR − incidence in desensitization regimens − but might also be of therapeutic value in the setting of manifest high-risk ABMR as indicated by detection of C1q-binding DSA.

## Materials and Methods

### Patients, Immunosuppression, and Additive Treatment

All patients received standard immunosuppression at transplantation with calcineurin inhibitors, antimetabolites, and steroids. Patients received tacrolimus and basiliximab if they were pretransplanted or had an HLA (A, B, DR) mismatch higher than or equal to 3. All patients received prophylactic therapy with trimethoprim/sulfomethaxol and valganciclovir for pneumocystis jirovecii and CMV infection, respectively. In advent of ABMR, patients received IA with a targeted plasma volume of 8,000 mL. Refractory ABMR was given when a serological and histological diagnosis of ABMR was established and individual CNI target through level increase, IA, and steroid therapy was not effective. When C1q-binding antibodies were detected, DFPP + IA was initiated, and no single IA treatment was performed afterwards.

### Transplant Biopsies

Biopsies were taken when clinically indicated. Clinical indication was given in the advent of primary graft dysfunction, deterioration of graft function (e.g., serum creatinine rise or proteinuria, deterioration in excretory function), or at the treating physician's discretion during ABMR treatment. No protocol biopsies were obtained. Histomorphological examinations were carried out on formalin-fixed paraffin-embedded tissue according to standard methodology (including immunohistology for IgA, IgG, IgM, C1q, C3, fibrin, fibrinogen, C4d, CD68, SV40) and analyzed according to the revised Banff criteria 2017.

### Plasmatic Measurements and Detection of DSA

C1q, IgG, IgM, and fibrinogen levels were taken before and after DFPP + IA treatments. Serum samples for analyses of DSA were taken before and after treatment cycle was implemented. HLA reactivity was analyzed by Luminex single antigen assay according to the manufacturer's protocol.

### Rejection Treatment with DFPP + IA

Plasma was obtained by filtration (“P2dry” by Fresenius Medical Care, Bad Homburg, Germany) of ACD-A-anticoagulated blood (ACD-A/blood ratio 1:40–1:50) via shunt or Shaldon catheter at a blood flow rate of 120–150 mL/min. A fraction of 20% (24–30 mL/min) plasma was extracted and run through a fractionator (“Monet” MSC, ibidem). Filtrate then passed through a GAM-146-coated adsorber system (“Globaffin,” FSM, ibidem) and was reintroduced to the flow-through of the plasma filter to eventually meet the blood stream again. The adsorber system used here comprises two hemoperfusion cartridges, self-regenerating after 250 mL fractionated plasma was loaded. The self-regeneration is executed by a cascade of (sequentially) NaCl (70 mL total), glycinHCT pH 2.8 (150 mL total) for antibody cleavage, PBS + 0.01% NaN_3_ (150 mL total), and NaCl (150 mL total) rinsing steps at a flow rate of 60 mL/min. Every column was loaded 10 times per treatment; thus, 20 treatment/rinsing cycles were performed, which adds up to 5,000 mL of plasma treated in every session. A circuit diagram is provided in online supplementary Figure S1 (for all online suppl. material, see www.karger.com/doi/10.1159/000528748).

### Statistical Analyses

Most of the presented data are descriptive. With regard to statistical analysis of plasmatic measurements, Wilcoxon signed-rank test was performed. Statistical significance was assumed at a two-sided *p* ≤ 0.05.

## Results

Four patients with biopsy-proven ABMR with C1q-binding de novo DSA are reported. Two of those cases would be assigned to early ABMR within the first year after transplantation (diagnosis of ABMR at day 6 and 201), and two cases developed ABMR later (diagnosis of ABMR at day 360 and 1,152). All patients were treated with IA prior to combination treatment with DFPP + IA but remained refractory. The respective patient baseline characteristics and concomitant immunological therapies are depicted in Tables 1 and 2, respectively. All patients had graft dysfunction with rising plasma creatinine concentrations; the index early ABMR case was dialysis dependent for 4 weeks following renal transplantation. Combined treatment of DFPP + IA was associated with kidney function improvement and resolution of ABMR in the early cases but appeared to be of lesser therapeutic value the later ABMR occurs in allograft live.

### Case Series

#### Patient 1 with Early ABMR

**Fig. 1:**
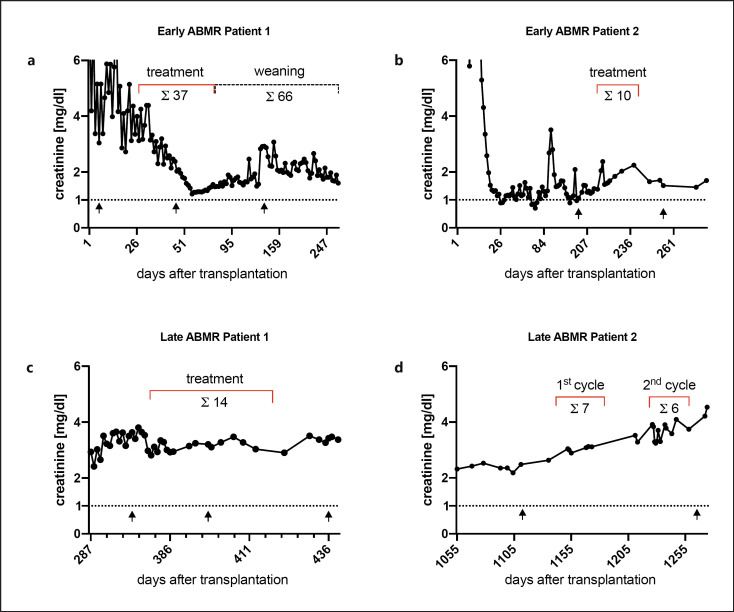
Depicted are the serum creatinine courses of the C1q-DSA-positive ABMR cases. “Treatment” refers to combined DFPP + IA treatments; “Σ” depicts total number of treatments in the respective treatment cycle. The arrows indicate allograft biopsies taken at respective days after transplantation.

A 62-year-old female received a second deceased-donor allograft. Persistent graft dysfunction prompted steroid boluses for the presumed diagnosis of cellular rejection. An allograft biopsy on day 6, however, showed ABMR. The patient received ATG for 21 days, as well as plasma exchange, followed by IA and infusion of high-dose polyvalent immunoglobulins (IVIG); however, creatinine and biopsy results did not improve. On a compassionate-use basis, we implemented DFPP + IA, which was associated with significant improvement in excretory kidney function and plasma creatinine decline in a timely fashion (Fig. 1a). Because we performed DFPP + IA for the first time, we decided to “wean” the patient over a period of 1 year, being concerned of humoral rebound effects following prompt discontinuation. Besides two uncomplicated urinary tract infections with associated and subsequently resolving acute allograft injury, the patient had a favorable outcome with a serum creatinine of 1.68 mg/dL at day 1,856 after transplantation and, importantly, no severe infectious or neoplastic complications.

#### Patient 2 with Early ABMR

A 36-year-old patient received a second deceased-donor allograft and experienced delayed graft function primarily attributed to prolonged cold ischemia time. However, first kidney biopsy on d7 showed humoral rejection, which resolved in a subsequent biopsy on d16 following IA (7 treatments) and anti-thymocyte globulin therapy (875 mg abs). The patient developed relapsing urinary tract injections, which supposedly triggered a second episode of humoral rejection within the first year of transplantation (d201). After detection of a C1q-binding de novo DSA, we initiated DFPP + IA for a total of 10 treatments (Fig. 1b). A follow-up biopsy showed marked improvement of transplant glomerulitis and peritubular capillaritis (Table 3). C1q-DSA levels declined and diminished eventually; non-C1q-binding DSA, however, persisted (Table 4). A concomitant CMV infection prompted antiviral treatment, which in combination with higher target tacrolimus trough concentrations led to improvement and stabilization of kidney function in the follow-up. The patient suffered from relapsing urinary tract infections due to reflux, which could be resolved by ureter neo-implantation. Allograft function remained stable with a serum creatinine of 1.86 mg/dL on day 1,592.

#### Patient 1 with Late ABMR

A 58-year-old patient received a living-donor allograft from his wife and was discharged with a serum creatinine of 1.8 mg/dL. During the first half year, he developed biopsy-proven BK virus nephropathy (serum concentration: 441k cop/mL) and systemic CMV reactivation (140k cop/mL). In the absence of BK-specific virustatic therapies, mycophenolate was discontinued and eventually replaced by leflunomide. CMV reactivation was treatment resistant, and subsequent virus sequencing revealed UL97 resistance mutation, which led to prolonged virus clearance under hyperimmunoglobulin treatment. Finally, BK and CMV clearance was achieved. At day 360 after transplantation, the patient developed ABMR with detection of a C1q-binding de novo DSA, which prompted DFPP + IA treatment (Fig. 1c). The patient received 14 combined treatments, which in spite of persistent MFI (Table 4 “late 1”) led to resolution of ABMR in the biopsy. Serum creatinine stabilized at 3.5 mg/dL throughout the following 3 years. Then, the patient, again, developed late ABMR, which eventually led to graft loss and re-introduction of dialysis treatment 5 years after transplantation.

#### Patient 2 with Late ABMR

A 44-year-old patient received a first allograft with stable graft function for the first two and a half years. At day 860, the patient developed ABMR and hence received plasma exchange. Subsequent biopsies showed no improvement and DSA was increasing. As a result of persistent DSA of 15k MFI on day 1,001, the patient received IA, which also did not improve the findings of subsequent biopsies. At day 1,135, we temporarily stopped IA due to nonclearing of humoral rejection and no significant reduction in DSA. Two weeks later, we detected C1q-binding de novo DSA, and at day 1,152, we prompted DFPP + IA (Fig. 1d). Despite significant reduction in C1q-binding DSA Cw4 after treatment with DFPP + IA, the C1q-binding DSA DQB1*06:03 persisted after the whole cycle (Table 4 “late 2”). We initiated a second cycle with DFPP + IA at day 1,226. After six treatments, we stopped DFPP + IA due to lack of clinical efficacy and progressive hypoproteinemia. Five years after transplantation, the graft was lost and the patient had to undergo dialysis.

### Plasmatic Measurements and Kidney Biopsy Results

**Fig. 2:**
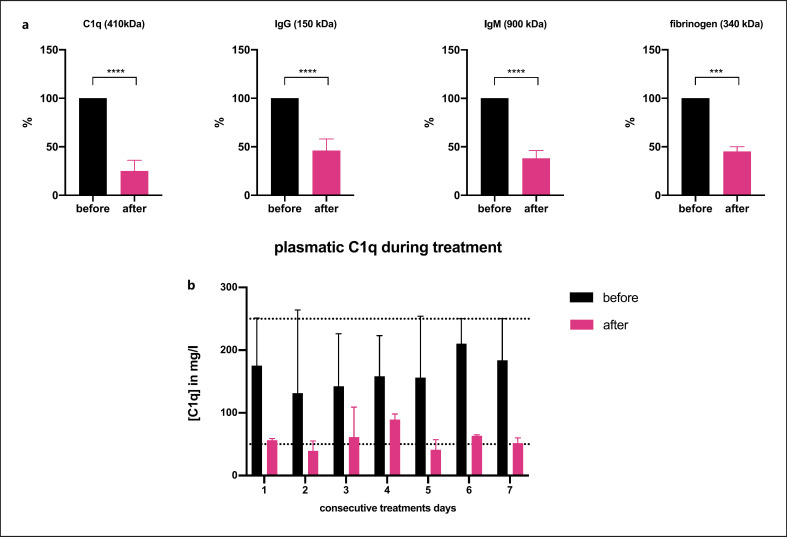
**a** Depicted are the absolute alterations of the respective molecules before and after a single DFPP + IA treatment. Index measurements (“before”) are normalized to 100%. **b** Total serum C1q levels before and after DFPP + IA treatments on seven consecutive treatment days. These data derive from patients “early 1” and “late 1.” The dashed lines mark the lower and upper border of normal C1q values. All data are presented as median + IQR. *** *p* < 0.001; **** *p* < 0.0001.

DFPP + IA proved effective in reducing macromolecular proteins as measured before and after treatment (Fig. 2a). Plasmatic C1q was reduced by 75% (median 25%, IQR 21–36% *p* < 0.0001, *n* = 39 measurements), IgM by 62% (median 38%, IQR 32–46%, measured only in one patient, *p* < 0.002, *n* = 10 measurements), IgG was reduced by 54% (median 46%, IQR 36–58%, *p* < 0.0001, *n* = 19 measurements), followed by fibrinogen with a median reduction of 55% (median 45%, IQR 38–50%, *p* < 0.0005, *n* = 12 measurements).

However, in a series of consecutive daily treatments, we did not find a sustained reduction in plasmatic C1q (Fig. 2b, *n* = 3 data points for every column). Baseline C1q levels before daily treatment did not differ according to consecutive days of treatment. In line with assumed rebound effects of gross plasmatic C1q and immunoglobulin concentrations, we did not find consistent reductions in DSA titers posttreatment (Table 4). In contrast, biopsy-derived ABMR evaluation showed reductions in inflammatory load and was interpreted as “improved” as compared to before taken biopsies for all cases but the second patient with late ABMR (Table 3).

### Complications

Frequently observed side effects were mild hypotension (drop of 10–30 mm Hg systolic blood pressure) during the course of treatment and mild thrombocytopenia afterward. Also, as ACD-A is a citrate-containing solution, patients occasionally developed clinically inapparent hypocalcemia and mild metabolic alkalosis. One patient experienced thrombosis of the vena cava superior due to repetitive jugular Shaldon catheterization, which eventually resolved after systemic anticoagulation. Naturally, one major effect of the DFPP + IA treatment in all patients was hypogammaglobulinemia, which was addressed by IVIG infusion (0.5 mg/kg body weight) after every third to fifth session, depending on serum values and clinical context. Concerning IVIG infusion, one patient developed a grade two anaphylactic reaction (according to the classification by Ring and Messmer), which successfully resolved following steroid and H1 antagonistic therapy. Subsequent IVIG infusions were uneventful under the respective premedication. Regularly, patients were substituted fibrinogen due to procedure-associated losses of serum coagulation effectors. Under these premises, no bleeding events were apparent.

## Discussion

The DFPP technique was primarily developed to treat patients with dyslipoproteinemia by clearing proteins such as apolipoprotein A1 and LDL-cholesterol; however, the membrane is not selective for lipoproteins. Instead, it could be demonstrated that a broad spectrum of high-molecular weight proteins are filtered, including fibrinogen, IgM antibodies, and complement factors. Therefore, DFPP is implemented in some centers in preconditioning regimens for antibody-incompatible kidney transplantation (AB0i [18] and/or HLAi [19]). Earlier, in a study by Pretagostini and others [20], DFPP was compared with IA for treatment of ABMR and found comparably both effective in resolving ABMR and safe. As demonstrated in the study by Eskandary et al. [17], a combination treatment of DFPP + IA appeared to be more effective in clearing IgM antibodies and complement factors, particularly C1q, compared to IA alone in a preconditioning regimen. In our study, reductions of the investigated macromolecules also declined posttreatment on a comparable level to those reported by Eskandary and others (medians, C1q 75% vs. 82%; IgM 62% vs. 69%, respectively). Reduction in IgG serum levels tended to be lesser in our study than that reported by Eskandary and colleagues (54% vs. 77%). Recently, Doberer et al. [21] published a case series of four highly sensitized kidney transplant recipients suffering from ABMR refractory to sole semi-selective IA. The pronounced capability of DFPP + IA in clearing main effectors of ABMR as compared to IA alone was the rationale to adopt this approach here in an experimental treatment intent. This study also reports on high-risk patients, here, those with C1q-binding de novo DSA. Our study supports the findings of Doberer et al. in that we also report favorable outcomes of treatment-refractory ABMR following DFPP + IA initiation in the early posttransplant period in high-risk patients. Interestingly, we did find stronger improvements in ABMR − severity, the earlier after transplantation rejection occurred. The immunobiology of humoral rejection is seemingly a function of time after transplantation [22, 23]; nevertheless, there is no contemporary theory addressing especially late active ABMR cases, where treatment often fails. In our patients, we did neither find sustained reductions in the MFIs of C1q-binding DSA nor marked baseline C1q level reductions following daily DFPP + IA treatment, both in resolved ABMR cases and those who did not respond to treatment. Rebound of systemic C1q and DSA here might be attributed to re-synthesis or re-equilibration. It is well established that the strength (or titer) of DSA is associated with risk of ABMR and severe tissue injuries. The correlation of DSA and clinical outcome, nevertheless, is not perfect, as there are patients that escape ABMR irrespective of DSA reduction with persistently high MFI. These findings highlight that improvements in allograft function following DFPP + IA in our study cannot be effortlessly associated with these measures and stress the necessity to refine our models of allograft rejection.

Our study has several limitations, most importantly its small sample size. This holds especially true for the surmised association of reduced DFPP + IA efficacy with transplantation vintage. Because patients were treated in a compassionate use intent, in principle, there was no control group. ABMR treatment in patients was not stratified. Especially the first patient received multiple immune-response-alternating treatments. Although we cannot rule out the impact of long-term effects of concomitant treatments in the reported cases, the chronological relationship of DFPP + IA initiation and renal recovery in the successful cases appears convincing. The study cohort, notwithstanding, remains somewhat heterogeneous with two patients having a second transplant and pre-DFPP + IA ABMR treatment being not strictly stratified, according mainly to infectiological complications. These circumstances, however, reflect clinical reality in transplantation medicine. Our data show a reduction in humoral immune effectors posttreatment on a single-treatment basis, but we did not find sustained DSA or C1q reductions in the long term, despite clinical and histological allograft improvement in three out of four patients. These findings suggest that MFI reduction alone in our patients is not sufficient to allow for allograft fate prognostication. The findings reported here have to be interpreted carefully. Frequently in (transplant) medicine, findings in smaller case series could not be confirmed in larger, stringently performed trials. In a meta-analysis of treatment options for ABMR in renal allografts, there is no or little evidence to support any treatment modality additive or instead of plasmapheresis and high-dose IVIG treatment [24]. Until now, there was no reasonably sized and stratified RCT conducted to address the efficacy of IA alone in ABMR since the first reports in 2001 [25] and 2007 [26]or a combination treatment of DFPP + IA or DFPP alone. The few small studies on DFPP with various additive immunomodulatory treatments in kidney ABMR reported conflicting results in heterogenous patient cohorts. In a recent report by Bennani and colleagues, DFPP plus rituximab did not result in clinical or histological improvement. However, in that study, acute and chronic active ABMR were mixed and patients received a maximum of 6 DFPP treatments. DFPP alone, nevertheless, is reportedly implemented in some centers as routine strategy for patients with ABMR, with some success [27]. The capability of DFPP + IA to clear blood immediately from humoral effectors is mechanistically obvious, scientifically reproducible, and can be considered established [28]. Whether that translates into improved allograft outcomes in ABMR in mid and long term has to be subjected to reasonably sized, controlled, and stratified RCTs, which unfortunately remain scarce in transplantation medicine.

## Conclusion

To our knowledge, this is the first report on DFPP + IA for C1q de novo DSA-positive treatment-refractory ABMR. It is the second report on DFPP + IA for treatment of ABMR as such. Our data show that DFPP + IA is comparably safe and might be considered a rescue treatment option for selected high-risk patients with proven severe acute humoral rejection in which other treatments failed.

## Statement of Ethics

The DFPP + IA treatment was implemented on a compassionate-use basis after written informed consent of the respective patients was provided. Both treatments are readily used in man. This research was carried out in accordance with the Declaration of Helsinki of the World Medical Association and complied with local guidelines for human studies. In this retrospective analysis, an ethics statement was not mandatory.

## Conflict of Interest Statement

The authors have no conflicts of interest to declare.

## Funding Sources

This research was not funded by external sources.

## Author Contributions

Martin Russwurm, Birgit Kortus-Goetze, Tanja Maier-Giebing, and Joachim Dirk Hoyer designed research. Hermann-Josef Groene analyzed renal biopsies. Philipp Russ collected and provided additional patient data. Martin Russwurm wrote the manuscript. Joachim Dirk Hoyer and Hermann-Josef Groene proofread the final manuscript.

## Data Availability Statement

All relevant data are reported in the article. Additional data can be provided by the corresponding author on request.

## Supplementary Material

Supplementary data

## Figures and Tables

**Table 1:** Baseline characteristics at transplantation

	Early ABMR patient 1	Early ABMR patient 2	Late ABMR patient 1	Late ABMR patient 2
Primary renal disease	Chron. GN/unknown	Reflux nephropathy	Hypertensive nephropathy	Alport syndrome
Recipient age in years	62	36	58	44
Number of prior transplants	1	1	0	0
Donor type	DD	DD	LD	DD
Cold/warm ischemia time	6 h 22' 24"	33 h 20' 43	3 h 38' 21"	10 h 2' 27"
Initial immunosuppression	Tac, MMF, CS, Blmab	Tac, MMF, CS, Blmab	CSA, MMF, CS, Blmab	CSA, MMF, CS, Blmab
HLA mismatch A/B/DR	0/1/1	0/0/1	0/1/2	1/1/1

Tac, tacrolimus; MMF, mycophenolate mofetil; CS, corticosteroids; Blmab, basiliximab; CSA, cyclosporin A; DD, deceased donor; LD, living donor.

**Table 2:** Immunological data at ABMR diagnosis and during DFPP + IA

	Early ABMR patient 1	Early ABMR patient 2	Late ABMR patient 1	Late ABMR patient 2
Day of ABMR diagnosis	7	201	370	1,152
IS at ABMR diagnosis	Tac, MMF, CS	Tac, MMF, CS	CSA, Lefl, CS	CSA, MMF, CS
CNI target through level	10–12 µg/L	10–12 µg/L	100–125 µg/L	75–100 µg/L
Antimetabolite dose	2 × 1,000 mg	2 × 750 mg	1 × 20 mg	2 × 500 mg
Prednisolone dose	20 mg	20 mg	10 mg	5 mg
ABMR treatment IA/No. of treatments	Yes/12	Yes/7	Yes/6	Yes/5
CNI target through level	10–12 µg/L	10–12 µg/L	150–175 µg/L	150–175 µg/L
ATG/mg abs	Yes/1,050	Yes/875	No	No
Steroid bolus (500 mg for 3 days)	Yes	Yes	Yes	Yes
IVIG/dose*	Yes/1 g/kg BW	No	Yes/1 g/kg BW	No
Rituximab	Yes	No	No	No
No. of total DFPP + IA sessions	103	10	13	14

All patients received IVIG at a dose of 0.5 g/kg BW as substitution therapy at DFPP + IA treatment cycles. CNI, calcineurin inhibitor; ATG, anti-thymocyte globulin; IVIG, intravenous immunoglobulin; IS, immunosuppression; BW, total body weight.

* Depicts high-dose IVIG treatment prior to DFPP+IA. All patients received IVIG at a low dose of 0.5 g/kg BW as substitution therapy at DFPP+IA treatment cycles.

**Table 3:** Renal biopsy histological analyses

Patient		g	i	ti	t	v	aah	cg	ci	ct	cv	mm	ptc	C4d	i-IFTA
Early 1	Pre	3	0	0	0	0	0	0	0	0	0	0	2	0	1
	
	Inter	2	0	0	0	0	0	0	0	0	0	0	1	0	x
	
	Post	1	0	3	0	0	0	0	3	3	2	1	1	0	3

Early 2	Pre	3	0	1	0	0	0	0	1	1	1	0	3	0	1
	
	Post	1	0	x	1	0	0	0	x	x	x	2	2	0	x

Late 1	Pre	3	0	0	0	0	0	0	3	3	0	0	2	0	3
	
	Inter	3	0	3	0	0	1	0	3	3	2	1	2	0	3
	
	Post	0	0	0	0	0	2	3	3	3	x	3	0	0	3

Late 2	Pre	0	0	0	o	o	3	2	1	1	0	0	3	3	0
	
	Post	1	1	2	1	0	2	3	2	2	2	1	2	2	0

Renal biopsy findings according to the revised 2017 Banff criteria. “x” depicts that the respective dimension could not be evaluated with regard to the sample obtained.

**Table 4:** Donor-specific antibody measurement during treatment

Patient	Measurement	Day	DSA	MFI
Early 1	Before	24	C1q-b. DPB1 * 04:02	**2,474**
	
	After	67	C1q-b. DPB1 * 04:02	**6,049**

Early 2	Before	196	DPB1 * 01:01 C1q-b. DPB1* 01:01 DPA1 * 02:01	17,274 **9,509** 18,287
	
	During cycle	230	DPB1 * 01:01 C1q-b. DPB1* 01:01 DPA1 * 02:01	16,908 **3,791** 17,639
	
	After	422	DPB1 * 01:01 C1q-b. DPB1* 01:01 DPA1 * 02:01	14,934 **neg** 15,685

Late 1	Before	379	C1q-b. DQA1 * 05:01 DQB1* 02:01	**24,707** 4,964
	
	During cycle	385	C1q-b. DQA1 * 05:01 DQA1 * 05:01	**24,337** 14,325
	
	After	433	C1q-b. DQA1 * 05:01 DQA1 * 05:01	**25,238** 8,349

Late 2*	Before	1,130	C1-b. Cw4 C1q-b. DQB1 * 06:03	**30,275** **26,115**
	
	In between	1,215	C1q-b. Cw4 C1q-b. DQB1 * 06:03	**1,123** **34,447**

“Before” and “after” refer to points in time before and after treatment with DFPP + IA. MFI, mean fluorescent intensity − threshold for DSA positivity was set at 1,000.

* In this patient, two cycles of DFPP + IA were implemented, “in between” refers to the depicted day between these two treatment cycles.
