# Supplementary material for: Efficacy of Double Membrane Filtration Immunoadsorption in Severe C1q-Binding Donor-Specific Antibody-Positive Acute Humoral Kidney Allograft Rejection: A Case Series
Source: Blood Purif. 2023 Apr 12;52(5):428–36. doi: 10.1159/000528748 (PMC10273878; doi:10.1159/000528748)
Supplement: Supplementary file 1 — Supplementary data [file bpu-0052-0428-s01.pdf]

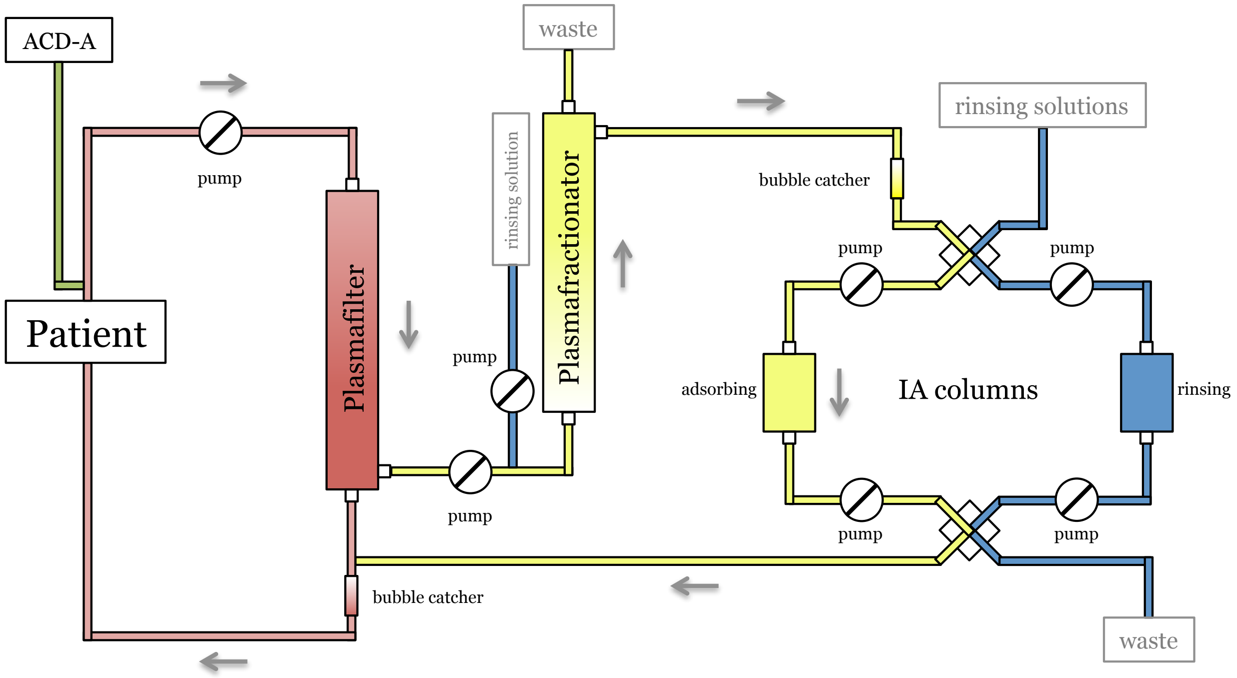

Supplementary Figure 1
Circuit diagram of [DFPP+IA]. For specifications of flow rate and extracted plasma fractions please refer to methods.
